# Prediction of Preterm Birth by Maternal Characteristics and Medical History in the Brazilian Population

**DOI:** 10.1155/2019/4395217

**Published:** 2019-09-25

**Authors:** Enio Luis Damaso, Daniel Lober Rolnik, Ricardo de Carvalho Cavalli, Silvana Maria Quintana, Geraldo Duarte, Fabricio da Silva Costa, Alessandra Marcolin

**Affiliations:** ^1^Department of Gynecology and Obstetrics, Ribeirao Preto Medical School, University of Sao Paulo, Ribeirao Preto, Sao Paulo, Brazil; ^2^Department of Obstetrics and Gynaecology, Monash University, Melbourne, Victoria, Australia; ^3^School of Clinical Sciences, Monash University, Melbourne, Victoria, Australia

## Abstract

**Objectives:**

The aim of this study was to assess the performance of a previously published algorithm for first-trimester prediction of spontaneous preterm birth (PTB) in a cohort of Brazilian women.

**Methods:**

This was a retrospective cohort study of women undergoing routine antenatal care. Maternal characteristics and medical history were obtained. The data were inserted in the Fetal Medicine Foundation (FMF) online calculator to estimate the individual risk of PTB. Univariate and multivariate logistic regression analyses were performed to determine the effects of maternal characteristics on the occurrence of PTB. A receiver-operating characteristics (ROC) curve was used to determine the detection rates and false-positive rates of the FMF algorithm in predicting PTB <34 weeks of gestation in our population.

**Results:**

In total, 1,323 women were included. Of those, 23 (1.7%) had a spontaneous PTB before 34 weeks of gestation, 87 (6.6%) had a preterm birth between 34 and 37 weeks, and 1,197 (91.7%) had a term delivery. Smoking and a previous history of recurrent PTB between 16 and 30 weeks of gestation without prior term pregnancy were significantly more common among women who delivered before 34 weeks of gestation compared to those who delivered at term were (39.1% vs. 12.0%, *p* = 0.001 and 8.7% vs. 0%, *p* < 0.001, respectively). Smoking and history of spontaneous PTB remained significantly associated with spontaneous PTB in the multivariate logistic regression analysis. Significant prediction of PTB <34 weeks of gestation was provided by the FMF algorithm (area under the ROC curve 0.67, 95% CI 0.56–0.78, *p* = 0.005), but the detection rates for fixed false-positive rates of 10% and 20% were poor (26.1% and 34.8%, respectively).

**Conclusions:**

Maternal characteristics and history in the first trimester can significantly predict the occurrence of spontaneous delivery before 34 weeks of gestation. Although the predictive algorithm performed similarly to previously published data, the detection rates are poor and research on new biomarkers to improve its performance is needed.

## 1. Introduction

Preterm birth (PTB), defined as a delivery that occurs before 37 weeks of gestation regardless of the newborn's weight [1], is a significant cause of death in children below the age of five and the leading cause of early neonatal morbidity and mortality, particularly in developing countries [2, 3]. Additionally, PTB accounts for more than half of the long‐term morbidity, especially among children born before 34 weeks of gestation [4, 5].

Although there are potential strategies for PTB prevention, such as cervical cerclage [6, 7] and administration of progesterone to patients with short cervix and/or history of PTB [8–11], significant declines in its rates have not been observed, which could be partially explained by the multifactorial etiology of PTB and by the inadequate selection of patients at increased risk [12].

In recent years, Bayes' theorem statistical models based on logistic regression or competing risks for the prediction of various pregnancy complications have been developed, allowing to estimate patient-specific probabilities through different combinations of maternal characteristics, medical history, and biomarkers, leading to better selection of high-risk women who could benefit from prophylactic interventions [13–15].

A recent large study has successfully validated a similar algorithm for the prediction of preterm preeclampsia and demonstrated that high‐risk patients benefit from low-dose aspirin [15, 16]. These combined predictive models often perform better than the use of history-based scoring systems that attribute similar weights to different maternal factors [17], but ideally should be validated in different populations and, when necessary, adapted [18].

Within this context, The Fetal Medicine Foundation (FMF) developed, in 2011, a model for the calculation of patient-specific risk of spontaneous PTB before 34 weeks of gestation that is applicable at 11–14 weeks of pregnancy [14]. Independent predictors used in this calculation are age, height, ethnic group, smoking, use of ovulation induction drugs, and history of PTB [14]. This model has not yet been validated externally, especially in developing countries such as Brazil.

The aim of this study was to assess the performance of the FMF predictive model for spontaneous PTB before 34 weeks of gestation in the Brazilian population.

## 2. Materials and Methods

### 2.1. Study Design and Data Collection

This was a retrospective cohort study of consecutive singleton pregnancies attending routine antenatal care, in a tertiary university hospital in Ribeirão Preto, Brazil, between February 2011 and February 2012. The previously mentioned risk factors for prematurity were investigated in a convenience sample of 1500 pregnant women recruited in Ribeirão Preto. The pregnant women included were evaluated in two different moments: prenatal (between 22 and 25 weeks) and at birth.

The study was approved by the local Ethics Research Committee (protocol number 300.231/2013) and reported according to the internationally acknowledged STARD criteria. Maternal characteristics and history were collected through the analysis of questionnaires, medical records, and telephone calls to the patients. The data were inserted in the FMF website to calculate the risk of PTB before 34 weeks of pregnancy [19]. Cases of PTB before 34 weeks were compared to those with birth after 37 weeks of pregnancy to determine the effects of maternal characteristics on the occurrence of PTB.

Gestational age was calculated from the last menstrual period (LMP) and confirmed by crown-rump length measurement at the time of the first-trimester scan. In case of discrepancy between the gestational age provided by LMP and ultrasound by more than seven days, the sonographic gestational age was used. The eligibility criteria included a singleton pregnancy at 11 + 0 to 13 + 6 weeks of gestation with a structurally normal fetus at enrolment. The exclusion criteria were the failure to acquire data from the questionnaires, medical records or through telephone calls, loss to follow up, miscarriage, intrauterine fetal death, the presence of major congenital anomalies at birth, and iatrogenic preterm delivery.

Pregnancy outcomes, including gestational age at delivery, occurrence of spontaneous preterm birth <34 weeks, and onset of labour (spontaneous onset, induction of labour or no labour) were collected from the patients' records and the risk calculation was performed by one of the investigators, who was blinded to the pregnancy outcomes.

### 2.2. Maternal Variables and Definitions

The primary outcome of the study was the occurrence of spontaneous preterm birth <34 weeks of gestational age. It was considered spontaneous early PTB all deliveries with spontaneous onset of labour and those with preterm pre-labour rupture of membranes leading to birth before 34 weeks of gestation. To calculate the risk of spontaneous PTB in the first trimester of pregnancy, we used the FMF online risk calculator available on the FMF website (https://fetalmedicine.org/research/assess/preterm) [19]. This model based on maternal factors alone is expected to detect 38.2% of the preterm deliveries in women with previous pregnancies at or beyond 16 weeks and 18.4% in those without, at a false positive rate (FPR) of 10% [19].

The following maternal variables were used to calculate the risk of spontaneous early PTB: age, ethnicity, height, mode of conception, smoking during pregnancy, and obstetric history. The risk was recorded in the database as a percentage.

In view of the high proportion of mixed ethnic backgrounds, it was necessary to use the following ethnicity classification in our population: White, Afro-Brazilian, Asian, and Mixed (Mulatto), as the equivalents of the ethnicity classification used in the original model (Caucasian, Afro-Caribbean, East Asian, and Mixed, respectively) [14], and there were no patients of South Asian background in our cohort. The obstetric history was categorized as follows: (a) no previous pregnancy (primigravida), (b) only previous gestations resulting in miscarriages at <16 weeks of gestation, (c) all of the previous pregnancies resulting in iatrogenic PTB (motivated by maternal and/or fetal complications), (d) previous history of a PTB between 16 + 0 and 30 + 6 weeks of gestation, (e) two PTB between 16 + 0 and 30 + 6 weeks of gestation, (f) a PTB between 16 + 0 and 30 + 6 weeks of gestation + another PTB between 31 + 0 and 36 + 6 weeks of gestation, (g) one PTB between 16 + 0 and 30 + 6 weeks of gestation plus at least one term delivery, (h) two PTB between 16 + 0 and 30 + 6 weeks of gestation plus at least one term delivery, (i) PTB between 31+0 and 36+6 weeks of gestation, (j) PTB between 31 + 0 and 36 + 6 weeks of gestation plus at least on term delivery, and (k) only deliveries at term.

### 2.3. Statistical Analysis

Categorical variables were expressed in absolute numbers and percentages, and continuous variables in means and standard deviations.

Comparisons between the spontaneous early PTB group with those delivering at term were performed with χ^2^ test and Fisher's Exact test for categorical variables and with Mann–Whitney U test for continuous variables. Univariate and multivariate logistic regression analyses were used to examine the effect of variables contributing significantly to PTB <34 weeks of gestation, with reported odds ratios (ORs) and 95% confidence intervals (CI). For calculations of detection rates and false-positive rates and to determine the best cut-off value for PTB before 34 weeks, a receiver operating characteristics (ROC) curve was produced using the predicted risks. The statistical software package SPSS 25.0^®^ (IBM SPSS Inc., Chicago, IL, USA) was used for data analyses, and a significance level of 5% was adopted

## 3. Results

**Figure 1:**
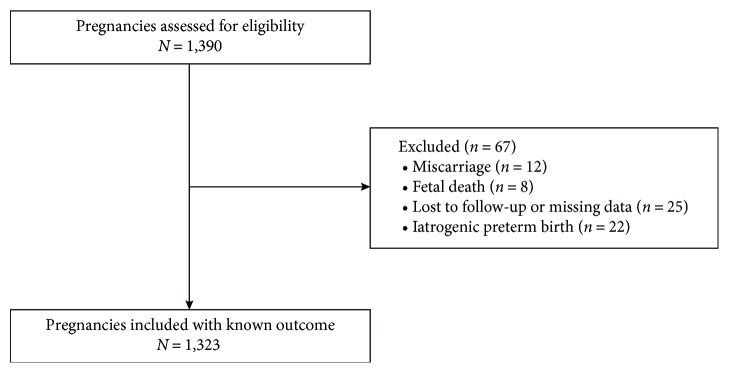
Study population flow diagram.

Of 1,390 pregnancies assessed for eligibility criteria, 67 (4.8%) were excluded, leaving 1,323 patients in the study (Figure 1). Of those, 23 (1.7%) had a spontaneous preterm birth before 34 weeks of gestation, 87 (6.6%) had a preterm birth between 34 and 36 + 6 weeks of gestation and 1,213 (91.7%) had a term delivery.

The demographic characteristics and the obstetric history of the study population are presented in Table 1. In the group of 1,323 analyzed patients, the mean age was 25.7 ± 6.0 years, and the mean height was 160.9 ± 6.3 cm, without significant differences between the groups. Significantly higher prevalence of smoking (39.1%) was observed in the group of delivery before 34 weeks of gestation (*p* = 0.001).

A previous history of preterm delivery without term deliveries was significantly more common among women delivering between 34 and 36–+–6 weeks of gestation, and the history of two premature births before 30 weeks of gestation without a prior term delivery was significantly more common in women who subsequently delivered before 34 weeks of gestation (Table 1). The average risks for preterm birth among women who indeed delivered before 34 and after 37 weeks' gestation were 1.3% and 0.7%, respectively.

**Figure 2:**
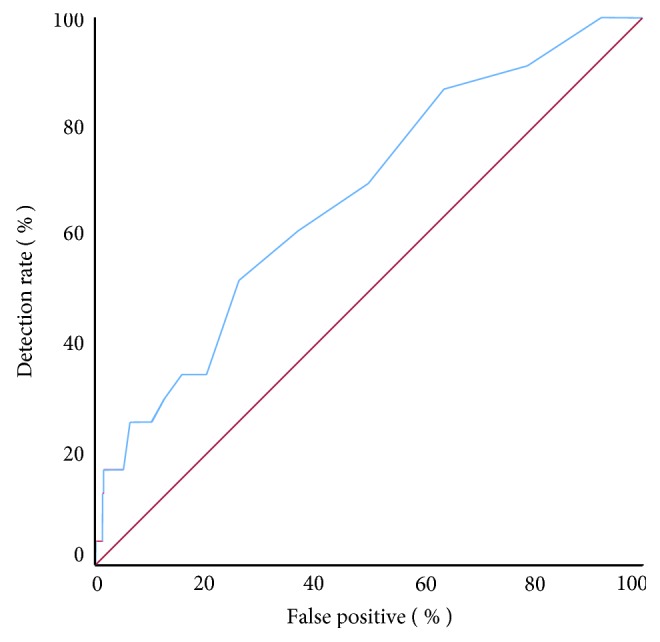
Receiver operating characteristics (ROC) curve for the prediction of premature birth before 34 weeks of gestation in the studied population.

Significant prediction of spontaneous preterm delivery before 34 weeks of gestation was achieved with the use of the FMF risk calculator, with an area under the curve (AUC) of 0.67 (95% CI0.56–0.78, *p* = 0.005), as shown in Figure 2. The risk cut-off with highest sensitivity and specificity was 0.75% (sensitivity = 60.9% and specificity = 63.2%). The detection rates were 26.1% and 34.8% for fixed false-positive rates of 10% and 20%, respectively.

Table 2 shows the influence of maternal risk factors (used in the risk calculator), in the form of odds ratios (OR), on the occurrence of PTB <34 weeks, after applying univariate and multivariate logistic regression analyses. Due to the small number of women with an obstetrical history of PTB in the studied population, it was considered as relevant obstetrical history the spontaneous and elective PTB at before 37 weeks of gestational age. Smoking and a previous history of spontaneous PTB increased the risk of a spontaneous PTB before 34 weeks of gestation. Pregnant women who smoked presented a risk of PTB had a fourfold increase in risk (adjusted OR 3.74, 95% CI 1.55–9.34, *p* = 0.005) when compared to women who did not smoke. Regarding the obstetric history, the risk of PTB was also nearly four times higher when the pregnant woman had a history of spontaneous PTB (OR 3.96, 95% CI1.08–14.56, *p* = 0.038) when compared to women with a history of term deliveries only. A previous history of spontaneous preterm birth was present in 17.4 fourfold increase in risk of the women who delivered before 34 weeks and in 3.8 fourfold increase in risk of those who delivered after 34 weeks. The other variables did not represent significant risk factors in the study population.

## 4. Discussion

The present study has demonstrated that, first, a predictive model for preterm birth can be used in the general obstetric Brazilian population and, second, that while a previous history of preterm delivery and smoking constitute strong risk factors for preterm birth, other previously described associations could not be reproduced in our cohort.

Previous studies have shown that the risk of spontaneous PTB increases with age [20] and decreases with maternal height [21], is higher in women of Afro-Caribbean and Asian ethnicities when compared to Caucasian women [22], in smokers [14, 23], and in those who used ovulation induction drugs [14]. The risk of PTB in women with a history of PTB is higher and ranges from 15% to 50% [20]. The risk increases with the number of previous preterm deliveries and with lower gestational ages in which these deliveries occurred [24]. In this study, only smoking and a previous history of extreme preterm birth with no previous term deliveries were significantly associated with birth before 34 weeks of gestational age. Smoking and previous preterm birth, each, increased by a factor of four the risk of spontaneous birth before 34 weeks of gestation. The discrepancies in relation to previous studies could perhaps be explained by the small number of premature deliveries in the present dataset and by differences in maternal characteristics, such as a low number of cases of assisted reproduction and a higher proportion of mixed ethnic background in our population, including women of various degrees of miscegenation. Diverse socio-economic conditions that are beyond age and racial origin can also lead to prematurity, constituting unknown confounders for the specific outcome of preterm birth [22, 25]. The rate of spontaneous PTB before 34 weeks of gestational age was 1.7%, slightly higher than the one reported in the original study (1.1%) [14], which is likely due to differences in maternal characteristics and a lower socio-economic status in the study population.

A previously published predictive model [14] was applied to determine the individual risk of preterm birth before 34 weeks of gestation in the Brazilian population, with results that were very similar to those published in the development of the algorithm by Beta et al. in 2011 (27.5% at a false-;positive rate of 10%), and the same area under the ROC curve of 0.67 [14].

Although significant prediction of spontaneous preterm birth before 34 weeks of gestation was provided by individual-risk calculation, the performance of this algorithm based on maternal characteristics and obstetric history alone is poor. Nevertheless, this could serve as a background risk in better predictive models that include the measurement of biochemical or biophysical markers, such as cervical length [9, 26]. The addition of biomarkers that prove to be different in women who are destined to deliver prematurely can potentially improve the screening performance and reduce false-positive rates in the near future. The predictive model performed better than dividing women into low- or high-risk groups based on the previous history of preterm birth alone, a risk factor that was present in only 4 of the 23 (17.4%) patients who delivered before 34 weeks of gestation and would not allow risk stratification in nulliparous women.

Additionally, the use of individual-risk calculation can lead to stratification of care, with reassurance of low-risk women and close monitoring of the high-risk group, or with the use of contingent sequential screening tests or preventive strategies, including more frequent antenatal visits, attenuation or abolition of other risk factors (such as smoking), and complementary investigation, reversing our current pattern of concentrated care in the last trimester [27]. Moreover, the risk calculation is inexpensive and easy to apply in the prenatal care in low-resource settings since maternal history collection is an integral part of prenatal care and the risk can be calculated online [19].

Higher accuracy of combined tests that estimate patient-specific risks over guidelines that rely on the previous history for the selection of high-risk groups has been reported in studies validating predictive models for other pregnancy complications [17, 28]. Such findings also indicate that, despite possibly requiring adjustments in different populations, robust predictive models developed with a large number of individuals can be externally validated and should be preferred over the development of new predictive models [18].

To the best of our knowledge, this is the first study to report the results of the application of a previously published predictive model for preterm birth in a Brazilian cohort. Its main limitations are the retrospective nature and the small absolute number of patients with preterm deliveries before 34 weeks of gestation or in certain groups of obstetric history. Another limitation is that, while the study was observational, patients with a significant history of preterm birth may have received increased surveillance or preventive measures, which would lead to lower performance of the algorithm. To minimize bias, however, the risk calculation was performed by one of the investigators who was blinded to the outcomes and, reassuringly, the algorithm performed as previously described.

In conclusion, the Fetal Medicine Foundation algorithm for the calculation of patient-specific risk performed as expected, with detection rates that are similar to those previously reported in the literature. Nonetheless, the discrimination of such a history-based model is poor. Effort should be made to identify potential predictive biomarkers that could result in improved detection of high-risk cases.

## Figures and Tables

**Table 1:** Baseline characteristics, obstetric history, and average risks for preterm birth before 34 weeks of the study population (Hospital das Clínicas, Ribeirão Pretro, São Paulo, Brazil, 2016).

Maternal characteristic	Term (*n* = 1, 123)	Preterm 34–37 w (*n* = 87)	*p* value^§^	Preterm <34 w (*n* = 23)	*p*value^§^	TOTAL (*N* = 1, 323)
Age (years), mean ± SD	25.6 ± 6.0	26.3 ± 6.1	0.31	27.1 ± 5.8	0.21	25.7 ± 6.0
Height (cm), mean ± SD	161.0 ± 6.3	160.1 ± 6.1	0.23	160.7 ± 6.3	0.84	160.9 ± 6.3
Ethnicity						
Caucasian, n (%)	628 (51.8)	45 (51.7)	0.61	11 (47.8)	0.89	684 (51.7)
Afro-Brazilian, n (%)	139 (11.5)	8 (9.2)		2 (8.7)		149 (11.3)
Mixed, n (%)	442 (36.4)	33 (37.9)		10 (43.5)		485 (36.7)
Asian, n (%)	4 (0.3)	1 (1.1)		0 (0)		5 (0.4)
Smoking	146 (12.0)	15 (17.2)	0.16	9 (39.1)	0.001^∗^	170 (12.8)
Nonsmoking	1067 (88.0)	72 (82.8)		14 (60.9)		1153 (87.2)
Use of ovulation-inducing drug	16 (1.3)	16 (1.3)	0.62	0 (0)	1.00	16 (1.2)
Obstetric history						
Primigravidae, n (%)	541 (44.6)	35 (40.2)	0.43	8 (34.8)	0.35	584 (44.1)
Previous miscarriage(s) <16 w n (%)	80 (6.6)	4 (4.6)	0.46	3 (13.0)	0.20	87 (6.6)
Previous sPTB 16–30 w (one event), n (%)	2 (0.2)	2 (2.3)	0.02^∗^	1 (4.3)	0.06	5 (0.4)
Previous sPTB 16–30 w (two events), n (%)	0 (0)	0 (0)	—	2 (8.7)	<0.001^∗^	2 (0.2)
Previous sPTB 16–30 w (one event) plus sPTB 31–36 w, n (%)	0 (0)	0 (0)	—	0 (0)	—	0 (0)
Previous sPTB 16–30 w (one event) plus term delivery, n (%)	1 (0.1)	0 (0)	1.00	0 (0)	0.98	1 (0.1)
Previous sPTB 16–30 w (two events) plus term delivery, n (%)	0 (0)	0 (0)	—	0 (0)	—	0 (0)
Previous sPTB 31–36 w, n (%)	26 (2.1)	5 (5.7)	0.05	1 (4.3)	0.40	32 (2.4)
Previous sPTB 31–36 w plus term delivery, n (%)	13 (1.1)	1 (1.1)	1.00	0 (0)	1.00	14 (1.1)
Previous iatrogenic delivery <37 w n (%)	8 (0.7)	1 (1.1)	0.47	1 (4.3)	0.16	10 (0.8)
Previous term deliveries, n (%)	543 (44.8)	39 (44.8)	0.99	7 (30.4)	0.17	589 (44.5)
Risk of preterm birth <34 w (%), mean ± SD	0.76 ± 0.50	0.99 ± 1.16	0.39	1.37 ± 1.59	<0.01^∗^	0.78 ± 0.6

^§^ *p* value for each group when compared to term delivery; ^∗^Statistically significant at 0.05 significance level. SD: standard deviation; w: weeks of gestation; sPTB: spontaneous preterm birth. χ^2^ test and Fisher's exact test for categorical variables and by Mann–Whitney U test for continuous variables.

**Table 2:** Odds Ratios and 95% confidence intervals obtained by univariate and multivariate logistic regression analysis for spontaneous preterm birth <34 weeks of gestational age (Hospital das Clínicas, Ribeirão Pretro, São Paulo, Brazil, 2016).

Independent variables	Univariate Analysis			Multivariate Analysis		
	OR	95% CI	*p*value	OR	95% CI	*p*value
Maternal age (years)	1.038	0.969–1.112	0.283	1.032	0.960–1.110	0.391
Maternal height (cm)	0.995	0.929–1.066	0.892	1.005	0.939–1.075	0.891
Use of ovulation‐inducing drugs	1.687	0.090–31.653	0.726	1.268	0.532–23.105	0.872
Ethnicity						
Nonwhite	1.180	0.498–2.798	0.707	1.281	0.357–1.802	0.581
Smoking	**4.382**	**1.788–10.738**	**0.001^∗^**	**3.744**	**1.500–9.344**	**0.005^∗^**
Obstetric history						
No previous pregnancy >16 w	1.255	0.475–3.321	0.647	2.779	0.760–10.156	0.122
Spontaneous PTB <37 w	**4.110**	**1.172–14.410**	**0.027^∗^**	**3.962**	**1.078–14.564**	**0.038^∗^**
Iatrogenic PTB <37 w	7.078	0.856–58.535	0.069	6.386	0.718–56.785	0.096

^∗^Significant at 0.05 level. OR: odds ratio; CI: confidence interval; PTB: preterm birth; w: weeks of gestation.

## Data Availability

The data used to support the findings of the study “Prediction of preterm birth by maternal characteristics and medical history in the Brazilian population” are available from the corresponding author upon request.
